# *Tremella fuciform* Polysaccharides: Extraction, Physicochemical, and Emulsion Properties at Different pHs

**DOI:** 10.3390/polym15071771

**Published:** 2023-04-02

**Authors:** Lili Tian, Yrjö H. Roos, Laura G. Gómez-Mascaraque, Xu Lu, Song Miao

**Affiliations:** 1 Teagasc Food Research Centre, Moorepark, Fermoy, P61 C996 Cork, Ireland; 2 School of Food and Nutritional Sciences, University College Cork, T12 K8AF Cork, Ireland; 3 China-Ireland International Cooperation Centre for Food Material Sciences and Structure Design, Fujian Agriculture and Forestry University, Fuzhou 350002, China

**Keywords:** *Tremella fuciform* polysaccharides, molecular character, emulsion property, pH

## Abstract

The chemical composition, macromolecular characteristics, and structure of four types of *Tremella fuciform* polysaccharides (TPS) were analyzed, including one TPS that was extracted in the laboratory (L-TPS) and three commercial TPS. The effects of pH on the properties of TPS emulsions were investigated by analyzing their zeta potential, particle size, apparent viscosity, and stability. The results showed that L-TPS presented a higher percentage content of protein (2.33%) than commercial TPS (0.73–0.87%), and a lower molecular mass (17.54 × 10^6^ g/mol). Thus, L-TPS exhibited the best emulsifying activity but gave poor emulsion stability. The droplet sizes and apparent viscosity of commercial TPS-stabilized emulsions were larger or higher in acidic environments. At pH 2, the apparent viscosity was the lowest for L-TPS. Commercial TPS emulsions were most stable at pH 6, while the L-TPS-stabilized emulsion was most stable at pH 2. The obtained results revealed that the emulsifying properties of TPS varied and the effects of pH on emulsion characteristics differed, as determined from the molecular mass, macromolecular characteristics, and structure. This research is useful for expanding the application of TPS as a novel food ingredient in emulsions.

## 1. Introduction

Plant-based polysaccharides play an important role in emulsions. Compared to synthetic materials, they are renewable, biodegradable, biocompatible, and easily modified [1]. Different polysaccharides show different emulsifying and emulsion stabilization mechanisms. Some of them present a satisfying emulsification capacity because of their hydrophobic groups in the polysaccharide chains or the proteins inside. These polysaccharides, which are considered emulsifying agents, can provide substantial surface activity at the oil-water interface, and hence, they can reduce the interfacial tension and facilitate the formation of fine droplets during emulsification [2]. Some other polysaccharides maintain the stability of emulsions through the thickening effect, steric hindrance, or electrostatic interactions. Normally, these polysaccharides belong to emulsifying stabilizers [3]. No matter what they are, their emulsifying abilities depend on their molecular properties. It has been reported that the molecular mass, electrical charge, branching, hydrophobicity, or conformational flexibility of polysaccharides can determine their functionalities [4]. Chen et al. [5] studied the physicochemical properties of tea polysaccharide conjugates (gTPC-T) and their two separate fractions with average molecular weights of 989 kDa (gTPC-1) and 48 kDa (gTPC-2), respectively. Compared to gTPC-2, gTPC-1 showed higher emulsification stability, which was caused by the differences in the chain lengths and conformations of the polysaccharides. This was similar to the result of Li et al. [6], who showed that the emulsifying activity of polysaccharide conjugates (TPC) from Chin brick tea was negatively correlated with their molecular mass, while the emulsion stabilization of TPC was positively related to molecular mass. In addition to molecular mass, the structure of polysaccharides also affects the emulsifying properties [7]. In the research of Olawuyi et al. [8], the relationship between the pH values (4.2, 6.8, and 9.2) of the extraction process and the functional properties of okra leaf polysaccharides was evaluated. Polysaccharides extracted under pH 4.2 showed a rapid emulsion formation, which was contributed by their long side-chain branching, i.e., arabinan, galactan, and arabinogalactan. Except for these factors, different environmental conditions (e.g., pH, salt, and temperature) also affect the properties of polysaccharides before emulsion preparation, further changing the emulsion stability. For example, under low pH environments, the absolute value of the zeta potential of anionic polysaccharides is normally lower than that at other pHs. As a result, the decreasing electrostatic repulsion may lead to a poorer resistance of the polysaccharide-coated oil droplets to aggregation and particle growth [9]. Another effect of pH is that it can alter the morphology of polysaccharides [10]. Xu et al. [11] showed that alginate had a higher negative charge density than pectin on the emulsion droplets, but the emulsion made by the intermediate concentrations of pectin was more stable than the emulsion made by alginate at all pHs. This result was explained by differences in the porosity and thickness of the polysaccharide layer. It is therefore necessary to study and understand the molecular properties of polysaccharides before studying their functions as food ingredients.

*Tremella fuciform* is the fruiting body of basidiomycete fungus tremella, which is a kind of mushroom that is mainly cultivated in Asian countries [12]. It is usually used as popular dietary supplement or tonic owing to its high nutritive value, medical benefits, and excellent taste. With a low fat content and caloric value, it is a good source of polysaccharides, dietary fibers, proteins, and vitamins [13]. More importantly, it also has therapeutic value and is useful in enhancing immunity and preventing diseases or disorders, which is mainly attributed to the active polysaccharides that are in it. The polysaccharides of *Tremella fuciformis* (TPS) possess various bioactivities, such as hypolipidemic, antioxidant, anti-aging, anti-inflammation, and anti-diabetic activities [14]. TPS are a kind of acidic heteropolysaccharide and their primary structure has been reported as a linear backbone of (1→3)-linked-α-D-mannan residues with the side chains composed of β-Xylp-, Fucp-, and β-GlcAp-. Their molecular mass is in the range of 1.08 × 10^3^–4.55 × 10^7^ g/mol, and mannose, fucose, xylose, glucose, glucuronic acid, and galactose are the predominant monosaccharide units [15]. Up to now, a lot of research on the biological activities of TPS has been reported [15], while there are only a few studies regarding their functional properties.

Recently, some commercial TPS have been used in cosmetics, while their applications as food ingredients are very limited. Zhang et al. [16] once proved that one type of commercial TPS showed better emulsifying stability compared with lotus seed polysaccharides, purple sweet potato, and gum Arabic in oil-in-water emulsions. The droplet size of the emulsion made by TPS showed fewer changes even with various pHs (pH 3.0, pH 6.5, and pH 10) and temperature (100 °C) treatment. This was contributed by both their negative charges and high viscosity property. Hou et al. [17] studied the emulsifying properties of one commercial TPS and proved that TPS illustrated better storage and freeze-thaw stability than gum Arabic, pectin, and carboxymethyl cellulose. Both the polysaccharide concentration and pH had effects on their emulsifying properties. Although TPS have been viewed as a potential ingredient in the emulsion system, only crude commercial TPS were chosen in these two previous studies and whether the other compositions that were inside had an effect on the properties of TPS was unknown. Whether the relationship between the pH and the properties of TPS-based emulsion varies with the type of polysaccharide was not mentioned. More importantly, the relationship between the emulsifying ability, molecular characteristics, and structure has never been studied; the mechanism of TPS acting as an emulsifier or stabilizer was unclear. 

Thus, in this research, TPS were extracted and purified in our laboratory. The molecular mass, monosaccharide composition, other chemical compositions, primary structure, and emulsifying properties of TPS were then studied and compared with another three types of commercial TPS. After that, the properties of these TPS-based emulsions were evaluated under different pH conditions by measuring their zeta potential, particle size, apparent viscosity, and stability. Here, we provided a guidance basis for expanding the application of TPS in the food industry field.

## 2. Materials and Methods

### 2.1. Materials and Reagents

The dried *Tremella fuciformis* was obtained from Gutian, Fujian, China. They were used to extract *Tremella fuciformis* polysaccharides in our laboratory, named L-TPS. For comparison, another 3 types of commercial polysaccharides (TMS, TPS-120, and TPS-160) were purchased from Shandong Freda Biological Co., Ltd. (Same raw material was used to extract these commercial polysaccharides. They were extracted and deproteinized with the same method and then subjected to degradation by enzyme and alkali solutions to obtain polysaccharides with different molecular masses). The sunflower oil was purchased from the Aldi supermarket in Ireland. The pectin from citrus peel and carboxymethylcellulose sodium salt were purchased from Sigma Chemical Co. (St. Louis, MO, USA). The soluble soybean polysaccharide was provided by Zhengzhou Tianshun Food Additives Co., Ltd. (Zhengzhou, China). All other chemicals and solvents were of analytical grade.

### 2.2. Extraction of Polysaccharides 

L-TPS was extracted and purified according to the previous method with some modifications [18]. The dried *Tremella fuciformis* was firstly washed and submerged into distilled water with the ratio of 1:40 (g/g) for 60 min at room temperature. After that the mixture was blended by a blender (Desire Matte Black Jug Blender, Russell Hobbs, England) for 7 min at the second gear. Then, the slurry was stirred and heated for 120 min in the water bath (90 °C). After being cooled, the extract was centrifuged at 6000× *g* for 20 min at 25 °C (LYNX 6000 Centrifuge, Thermo Fisher, Germany), and then the supernatants were collected and mixed with four volumes of alcohol (99.5%). The mixture was kept for 24 h at 4 °C, and then centrifugation was carried out (4000× *g*, 20 min, and 4 °C) to obtain the precipitate. The crude polysaccharides were then de-proteinized by Sevage method [19], and dialyzed and lyophilized to obtain the final polysaccharides (L-TPS).

### 2.3. Chemical Composition Analysis 

Phenol-sulfuric acid method was used to measure the total sugar content with D-glucose as the standard [20]. The protein content was estimated by Kjeldahl procedure as described in ISO 8968-1 [21]. Water content was determined using the vacuum-drying method (105 °C, 5 h) and the ash content was evaluated in duplicates by a muffle furnace [22,23].

The molecular mass of the polysaccharides was estimated by the high-performance size exclusion chromatography (HPLC-SEC). The HPLC system consisted of a Waters 2695 separations module and a Waters 2414 refractive index detector (Waters, Milford MA, USA). An OHpak SB-806 HQ column (Shodex, Japan) was applied to separate. The mobile phase was 10mM Na2HPO4-NaH2PO4 solution containing 150 mM NaCl (pH 7.4). Pullulan standards (P-82 kit, Shodex, Japan) were used to calibrate. The samples or standards were dissolved at 1 mg/mL in the mobile phase and filtered through 0.45 μm pore syringe filters (Sartorius, Germany). The SEC column and the detector were equilibrated at 40 °C. A volume of 20 μL of each sample/standard solution was injected and run at a flow rate of 0.5 mL/min. The analysis was performed in duplicates.

Before monosaccharide composition analysis, trifluoroacetic acid (2 mol/L) was used to hydrolyze TPS, which was then dried with nitrogen flow. Upon completion of hydrolysis, samples were washed with methanol and dissolved with 1 mL of water. Analysis of monosaccharide derivative was performed on a 7890B Gas Chromatograph equipped with an FID detector. Separation was carried out on the Agilent HP-5 column, with N^2^ as the carrier gas (1 mL/min). Sample (1 μL) was injected with a split ratio of 20:1. The temperature program was as follows: initial oven temperature 160 °C held for 2 min, then raised to 240 °C at 2 °C/min, and maintained at that temperature for 5 min (final hold time).

### 2.4. ATR-FTIR Spectroscopic Analysis

The FTIR spectra of TPS were recorded on an ATR-FTIR (Bruker, INVENIO 100453) at room temperature in the wavelength region between 4000 and 700 cm^−1^. Using Bruker Opus 5.5 software, spectra were obtained from 100 scans of each sample with an instrument resolution of 4 cm^−1^. 

### 2.5. Emulsifying Properties of TPS

Emulsifying activity (EAI) and stability indexes (ESI) of TPS were determined by the previous method [24]. The only difference was that the concentration of TPS solutions in this research was 1 mg/mL.

### 2.6. Preparation of TPS Emulsions

The dispersions of TPS (1 mg/mL) were stirred for 4 h at room temperature using a magnetic stirrer. pH values (2, 4, 6, 8, and 10) of dispersions were then adjusted with 0.1 M HCl and NaOH. After that, sunflower oil (10%, *w*/*w*) was added into TPS solutions and these mixture solutions were stirred for 1 min. Then, the resultant solutions (227.8 g) were mechanically sheared by a T25 ULTRA-TURRAX^®^ homogenizer (IKA, Germany) with a 25 mm head. The operation parameters were as follows: homogenizing speed, 12000 rpm; homogenizing time, 240 s.

### 2.7. Effect of pH on Emulsion Properties of TPS

#### 2.7.1. Zeta Potential and Particle Size 

Based on laser Doppler velocimetry at 25 °C, zeta potentials of the TPS emulsions were determined with the Zetasizer Nano-ZS (Mastersizer X, Malvern Instrument Ltd., Worcestershire, UK). Before analysis, TPS emulsions were diluted 20 times and stirred for 10 min. Master Sizer 3000 (Malvern Instruments Ltd., Worcestershire, UK) was used to analyze the droplet size distribution. Samples were diluted 10 times and added to an automated wet dispersion unit. The obscuration reached between 5 and 12%. 

#### 2.7.2. Apparent Viscosity

Apparent viscosity was measured by an ARG 2 rheometer (TA Instruments, Crawley, UK). The aluminum parallel plate was 60 mm in diameter, and the gap was 0.5 mm. Steady flow tests were performed at 25 °C. The shear rate varied from 0.1 to 100 s^−1^. For each test, about 2.5 mL of TPS solutions or emulsions were loaded in the center of the Peltier plate and allowed to equilibrate for 3 min before testing. Except for the steady shear flow curves, power-law model (Equation (1)) was also used to characterize the flow behavior of TPS solutions or emulsions.

(1) $$\tau =K{\dot{\gamma }}^{n}$$

where τ is the shear stress; K represents the consistency index (Pa·s^n^); $\dot{\gamma }$ represents the shear rate; and n represents the flow index.

#### 2.7.3. Emulsion Stability 

The stability of the emulsions was measured with the method reported by Klein et al. [25] with the same instrument (Lumifuge, LUM GmbH, Berlin, Germany). In this research, wavelength was 865 nm and samples were centrifuged at 3000 rpm for 1 h with transmission profiles measured at 1 min intervals. Sepview 4.1 (L.U.M GmbH) software was used to analyze the separation behavior.

### 2.8. Statistical Analysis

Each experiment was carried out in triplicates. Data obtained from three independent experiments were presented as the mean value ± standard deviation (SD). One-way analysis of variance (ANOVA) was performed by SPSS statistics 13.0 (IBM SPSS Statistics 27.0). *p* value between samples less than 0.05 were considered significant. All figures were plotted by GraphPad Prism9 software.

## 3. Results and Discussion

### 3.1. Chemical Characterization of TPS

For the commercial TPS, no significant differences were found in their chemical compositions. However, L-TPS and commercial TPS showed different results (Table 1). Compared with commercial TPS, L-TPS had lower content of total carbohydrates and higher content of protein (*p* < 0.05). These differences could be attributed to the different origins and culture conditions of the *Tremella fuciformis* [26]. In the research of Li et al. [26], two types of TPS were extracted with a similar extraction method from *Tremella fuciformis*, which was cultivated in two regions of China. Their polysaccharide contents were 88.46 ± 5.20% and 85.13 ± 7.13%, and protein contents were 2.26 ± 0.20% and 2.41 ± 0.13%, respectively. Other reasons resulting in composition differences among these TPS were related to the different extraction and purification processes. Different from L-TPS, commercial TPS were extracted in a pilot scale, and the proteins were removed by membrane filtration technology. 

The proteins detected in L-TPS evidenced that the proteins inside could not be completely removed by the Sevage method, especially for those conjugated to TPS [27]. The yield of L-TPS (6.66%) was higher than that reported by Liu et al. [28], who extracted TPS using hot water with a yield of 2.96%. This might be attributed to the high speed of shearing by the blender used in our research. It could break the cell walls, accelerate the dissolution of organics in cells, and thus, improve the yield of polysaccharides. Thus, to obtain a higher yield, it is necessary to improve the technology in the extracting and purifying steps. In terms of other components, higher moisture content was observed in L-TPS compared to commercial TPS, while no significant difference in the ash content was found among these TPS (*p* > 0.05). 

Usually, molecular mass and its distribution are important factors affecting the physicochemical properties of a polymer [29]. Diverse molecular masses were observed for these TPS (*p* < 0.05). TPS-160 showed the largest molecular mass followed by TPS-120, L-TPS, and TMS, respectively. Such differences might be due to the different origins of *Tremella fuciformis* and the extraction methods of TPS [30]. Similar results have been reported by Li et al. [26], who showed that the average molecular masses of two types of TPS were as high as 45.46 × 10^6^ g/mol and 25.98 × 10^6^ g/mol, respectively. Nevertheless, the molecular mass of TPS in the research of Wen et al. [31] was only 3.43 × 10^6^ g/mol. These differences might be attributed to the different *Tremella fuciformis* varieties or the determination methods. Different from TPS, the average molar masses of three polysaccharides fractions from *Pleurotus ostreatus* mushrooms were smaller than 0.14 × 10^6^ g/mol [32]. The polysaccharide from *Schizophyllum* had a molecular mass of 0.10 × 10^6^–0.20 × 10^6^ g/mol [33] and the molecular mass of lentinan was only roughly 0.50 × 10^6^ g/mol [34]. So, compared with other fungi polysaccharides, TPS have a relatively larger molecular mass, suggesting their potential as thickeners in the food system. This was because a larger molecular mass, longer molecular chain, and better intermolecular interaction of the polysaccharides would lead to more molecular entanglement [34,35].

Table 2 indicated that TPS were mainly composed of fucose, glucose, xylose, mannose, and glucuronic acid. In particular, mannose (32.07–44.91%) was the predominant monosaccharide of TPS, which was followed by glucuronic acid (12.85–25.38%), fucose (13.17–20.00%), xylose (14.92–18.08%), and glucose (3.31–14.98%), respectively. This observation was in good agreement with the previous work, which had proved that mannose was a repeating monosaccharide in the TPS and the mannose backbone was linked with side chains containing fucose, glucuronic acid, and xylose, as well as a small amount of glucose [36,37]. Glucuronic acid was thought as the main monosaccharide carrying negative charges in TPS. In comparison to commercial TPS, L-TPS showed higher glucuronic acid and glucose ratios, but lower mannose and fucose ratios (*p* < 0.05). Differences in the ratios of galactose and galacturonic acid were also observed but they were not significant.

### 3.2. ATR-FTIR Spectroscopy

**Figure 1:**
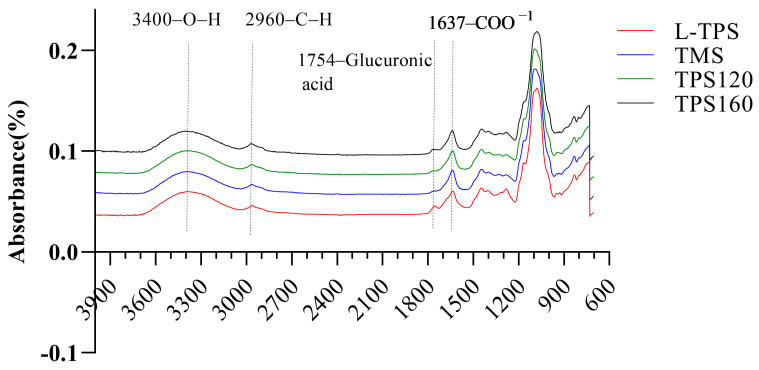
ATR-FTIR spectra of TPS.

The ATR-FTIR spectroscopy measurement of TPS samples was used to compare their chemical composition and structure. From this result (Figure 1), it can be seen that the ATR-FTIR spectra of TPS showed a broad peak of hydroxyl stretching at 3700–3100 cm^−1^, and stretching of aliphatic C–H bonds at 2960 cm^−1^ [38]. The next band in the region of 1754 cm^−1^ was possibly due to the glucuronic acid [39], and the peaks around 1637 and 1418 cm^−1^ were due to the asymmetric and symmetric stretching modes of the COO− group [40]. Detailed peak positions and assignments of each TPS were limited to the specific bands in the range of 1200–850 cm^−1^. In this region, peaks appeared with the presence of characteristics of glucan molecules (1032–1186 cm^−1^), which involved C–O stretching [41]. The band at 870 cm^−1^ was a characteristic absorption peak of mannose [42]. Such result was consistent with De Baets and Vandamme [43], who have proved that TPS had α-D-mannose in the main (or backbone) chain, and β-D-gluconic acid, β-D-xylose, and β-D-xylobiose linked to the C−2 of the main chain mannose. Among these TPS, only one difference was detected, as the peak intensities at 1754 cm^−1^ for L-TPS and TPS-160 were higher, suggesting structural differences between these two TPS and the other two.

### 3.3. Emulsifying Properties of TPS 

**Figure 2:**
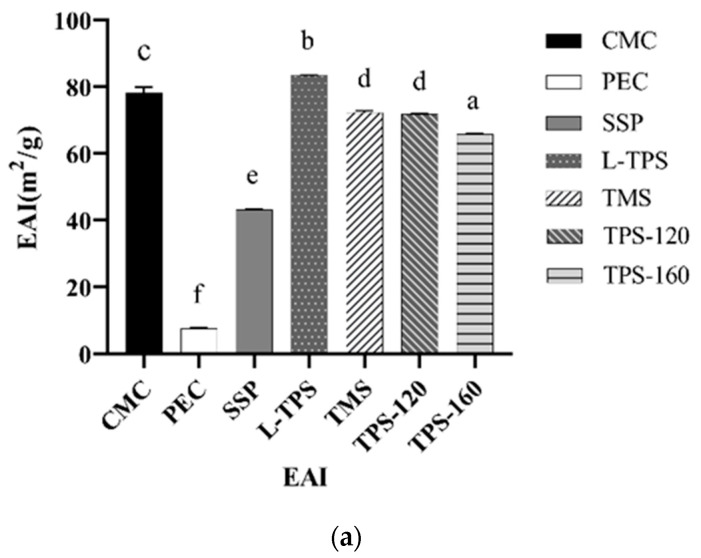

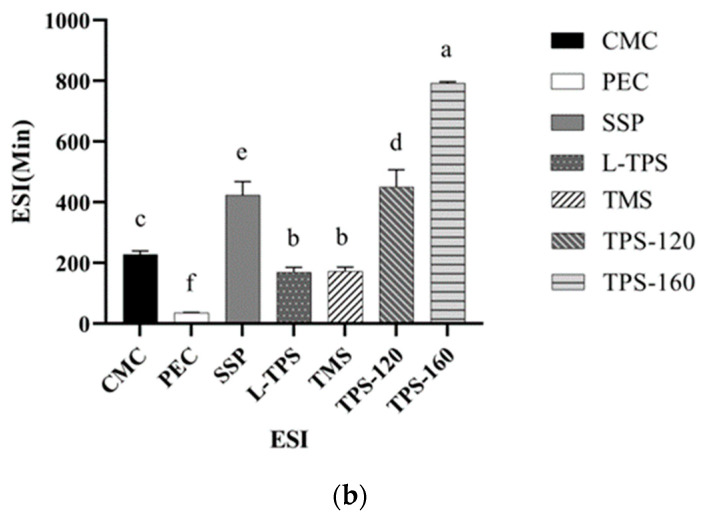
Emulsifying activity (**a**) and stability (**b**) of TPS. The concentration of these polysaccharides solution was 1mg/mL. Data represent the mean values of three replicates with error bars indicating the standard deviation. Different letters indicate significant differences (*p* < 0.05).

The EAI and ESI are the most commonly used indexes calculated by turbidity for evaluating the emulsifying properties of tested samples. Immediately after homogenization, the emulsions exhibited different turbidity values depending on the type of polysaccharides (Figure 2a). As expected, TPS showed a greater emulsifying ability compared with the pectin and soluble soybean polysaccharides. Such differences were related to their different backbones, chemical compositions (monosaccharide, water, and glucuronic acid contents), and functional groups (e.g., protein) [44]. Among these TPS emulsions, L-TPS exhibited the highest EAI value (84.46 m^2^/g), followed by TMS (72.41 m^2^/g), TPS-120 (71.98 m^2^/g), and TPS-160 (65.84 m^2^/g). This was probably attributed to the higher content of protein in L-TPS, which could easily absorb onto the surface of the oil drops with their hydrophobic groups [45]. Another possible reason was that L-TPS with low molecular mass could diffuse at higher rates to the water/oil interface [46]. For the commercial TPS, the EAI value of TPS-160 was lower than TPS-120 and TMS, while no significant difference was detected between TPS-120 and TMS. In this sense, molecular mass was not supposed to be the only factor affecting the emulsifying ability of commercial TPS in the research.

Different from the result of EAI, TPS-160 showed the highest ESI value (793 ± 4 min), followed by TPS-120 (450 ± 56 min), SSP (424 ± 44 min), and others (Figure 2b). This indicated that the use of TPS-160 led to a less opaque (turbid) but more stable emulsion compared with other types of TPS (L-TPS, TMS, and TPS-120). For the commercial TPS, their ESI values increased as their molecular masses increased. The molecular mass of TMS was smaller than L-TPS, whereas no significant difference in the ESI value was detected between them. By contrast, the ESI values of some other types of plant polysaccharides in previous reports were lower. For instance, the emulsifying stability of 5 to 20 mg/mL of Thunb leaves polysaccharides was only 37.46, 47.50, 48.79, and 53.64 min, respectively [47]. At 20 mg/mL, the emulsifying stability of four different polysaccharide fractions of peony seed dreg polysaccharides was only 39.66, 50.57, 41.67, and 30.96 min, respectively [48]. Similarly, the ESI value of pea pod polysaccharides in previous research [24] was 67.64 min. Therefore, it could be inferred that TPS, especially TPS-160, have more potential to stabilize o/w emulsions. Droplet size, viscosity, and steric or electrostatic repulsions of polysaccharides coated outside are the main characteristics of an emulsion system [49]. To further understand the properties of emulsions coated with TPS, the zeta potential, particle size distribution, apparent viscosity, and stability of these emulsions were thereby evaluated under different pHs. 

### 3.4. Effect of pH on Emulsion Properties of TPS

#### 3.4.1. Zeta Potential and Particle Size 

**Figure 3:**
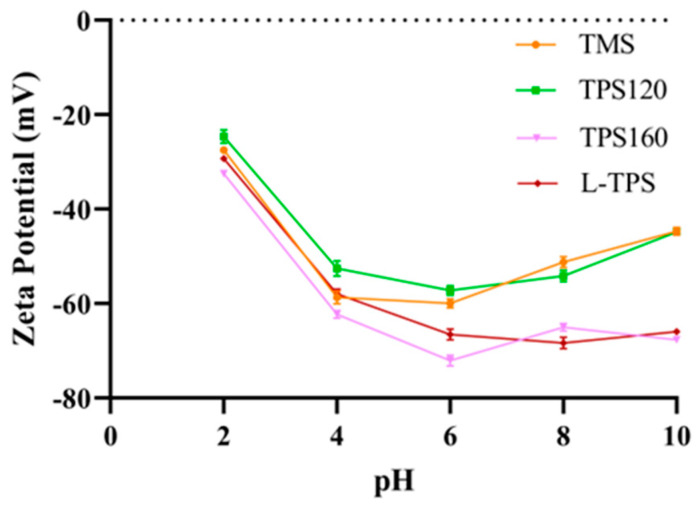
Zeta potentials of TPS emulsions with pH in the range of 2–10. Data represent the mean values of three replicates with error bars indicating the standard deviation.

The zeta potentials of all TPS emulsions with a pH in the range of 2–10 are shown in Figure 3. All emulsions showed negative zeta potential values in this pH range, suggesting that electrostatic repulsion existed to stabilize the oil droplets in these emulsions. The sharp drop in the zeta potential from pH 2 to pH 4 was attributed to the pKa of the glucuronic acid resident (near pH 3–4) [50]. Some differences could be found among these emulsion samples. Obviously, the zeta potentials of all TPS emulsions decreased with the pH increasing from 2 to 6. After that, the zeta potentials of the L-TPS and TPS-160 emulsions did not change a lot (around −72 mV), while TMS and TPS-120 emulsions increased slightly. As a result, the magnitudes of the zeta potential (absolute value) of L-TPS and TPS-160 were higher compared to the other two TPS. Such differences could be ascribed to the pH and polysaccharide type in the solution, which were the main factors affecting the electrical charge on oil droplets [51]. 

**Figure 4:**
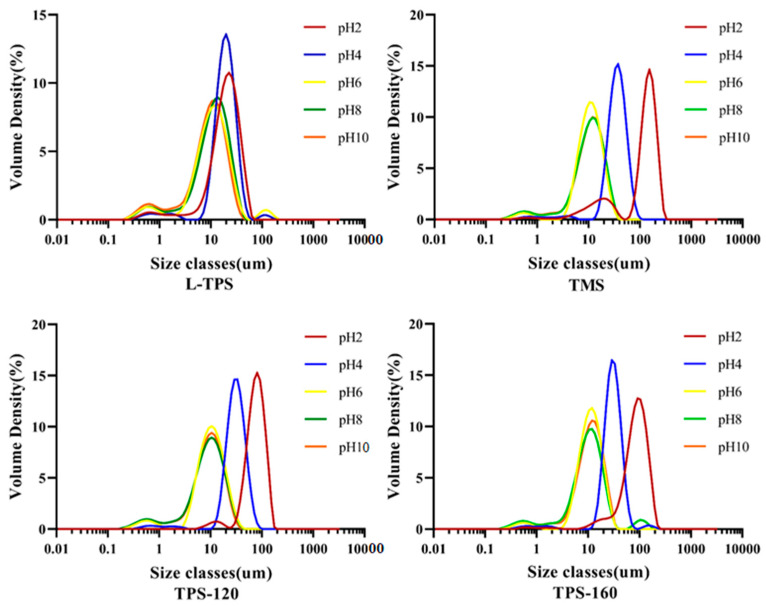
Particle size distribution of TPS emulsions.

Immediately after homogenization, these emulsions exhibited different droplet sizes depending on the pH of the aqueous phase (Table 3); the volume-weighted mean size (d_4,3_) values of these emulsions fluctuated from 10.1 µm to 125.00 µm; and the surface-weighted mean size (d_3,2_) values changed from 3.50 µm to 58.70 µm. Apparently, each emulsion at pH 2 and pH 4 showed bigger d_3,2_ and d_4,3_ values compared to those under higher pH conditions (*p* < 0.05). A remarkable left shift of the peaks of particle size distribution could be observed for all the TPS samples as the pH values increased (Figure 4). This result was in agreement with the behavior of polysaccharide conjugates from low-grade green tea, as reported by Han et al. [52]. In their research, with the pH increasing from 2.0 to 6.0, d_3,2_ of the emulsion decreased from 6.8 to 3.5 μm and remained relatively constant at about 3.0 μm from 7.0 to 8.0. Similarly, the particle size of the soy hull polysaccharide solution was larger than 40 nm at low pH 2–4, resulting in polysaccharide aggregates [53]. One possible reason was that compared to acidic conditions, higher repulsive forces under alkaline conditions could keep oil droplets stable [54]. Another reason was that polysaccharides became less stretched and had a markedly higher interfacial pressure when at low pH environments, which easily led to aggregation [54]. Compared to commercial TPS, the diameters of emulsions made by L-TPS were smaller under pH 2 and pH 4. As reported by Osano et al. [55], both proteins inside and the molecular mass were likely to play a significant role in the adsorption of polysaccharides. With a higher content of protein, the interfacial tension could possibly be decreased by the high amount of acetyl groups [56]. The lower the interfacial tension, the greater the extent to which droplets can be broken up during intense shearing, resulting in smaller emulsion droplets [2]. In addition, molecular mass was the relevant factor in the emulsification capacity of polysaccharides, which has been discussed in the Section 2.3.

#### 3.4.2. Apparent Viscosity

**Figure 5:**
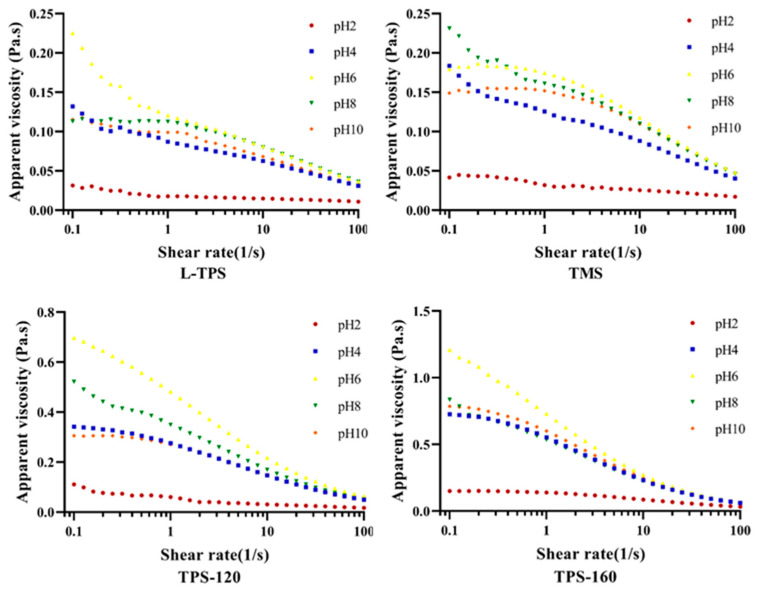
Apparent viscosity of homogeneous TPS solutions as a function of the shear rate at T = 25 °C.

**Figure 6:**
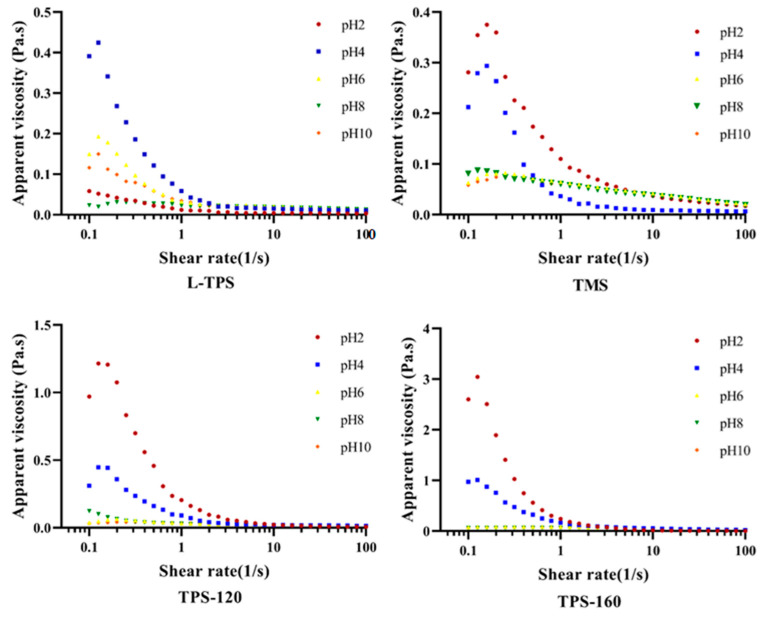
Apparent viscosity of TPS emulsions as a function of the shear rate at T = 25 °C.

**Figure 7:**
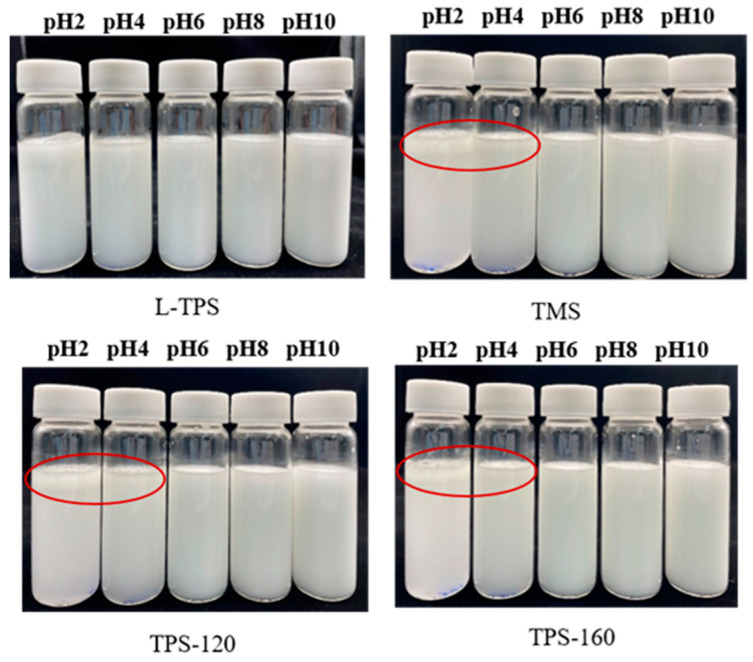
The appearance of TPS emulsions after 30 min at room temperature. The red circles represent the appearance of emulsion destabilization.

The apparent viscosity value of the emulsions is determined by composition of the interface layer and the interaction between droplets, which is an important indicator of emulsion characteristics [57]. To further understand how the viscosity property of TPS changes on the surface of oil drops under different pHs, the apparent viscosities of homogeneous TPS solutions were also evaluated (Figure 5). The results showed that all the samples were non-Newtonian fluids with a flow index (n) smaller than 1 (Table 4). The regression coefficient (R^2^) values indicated that the experimental data fitted well with the power-law, except for the TMS emulsion at pH 2. The homogeneous TPS solutions exhibited the lowest consistency index (K) values and highest n values at pH 2 (*p* < 0.05). This suggested that the TPS solutions at this pH possessed the weakest structures and exhibited more pronounced Newtonian-like behaviors compared to the other systems. A lower viscosity under extremely acidic environments might be caused by cleavage of the hydrogen bond, decrease in the molecular mass, and decomposition of the polysaccharides [58,59]. Different from homogeneous TPS solutions, all the commercial TPS emulsions at pH 2 showed the highest K values and lowest n values. More specifically, these emulsions exhibited the highest viscosity and strongest shear-thinning characteristics at pH 2, indicating a noticeable level in the initial structure (at zero shear rate) (Figure 6). Such results were consistent with emulsions stabilized by the soy hull polysaccharide [9]. This was because higher degrees of droplet flocculation happened at pH 2 and even at pH 4 [60], where non-adsorbed polysaccharides tended to increase the viscosity of the aqueous phase [55]. The phenomena of phase separation (Figure 7) and the larger d_4,3_ and d_3,2_ values in Section 3.4.1 could further prove this conclusion.

Different from commercial TPS, the L-TPS emulsion sample at pH 4 displayed the largest continuous phase viscosity, while at pH 2, the viscosity of the sample was the lowest. Protein residents might be the main reason for the lower viscosity value at pH 2 compared to pH 4. This was because, under low pH conditions, a strong interaction between polysaccharides and proteins might be formed with opposite charges and fewer polysaccharides aggregated in the continuous phase [61]. Despite the low viscosity, the L-TPS emulsion remained stable under pH 2, with no indication of phase separation during 30 min of storage at room temperature (Figure 7). This result further proved that L-TPS exhibited the best emulsion stabilizing capacity at pH 2. 

#### 3.4.3. Emulsion Stability 

**Figure 8:**
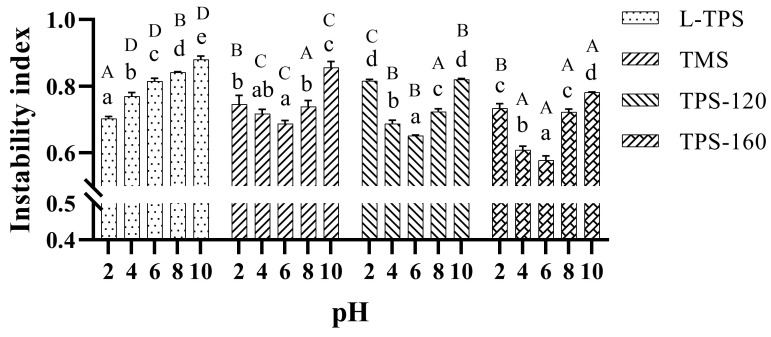
Instability index of TPS emulsions. Data represent the mean values of three replicates with error bars indicating the standard deviation. Different lowercase letters indicate significant differences in the same TPS sample at different pH values; different uppercase letters indicate significant differences in different TPS samples at the same pH value (*p* < 0.05).

Lumifuge is a technique allowing the separation process to be measured rapidly and thus gives a fast and accurate means of evaluating the emulsion dispersion stabilities. After treatment of centrifugation, noticeable differences in the degree of phase separation varied with the type of TPS and the applied pH (Figure 8). Apparently, such results were different from the discussions about emulsion’ stabilities in the above sections. This implied that the zeta potential, particle size, or apparent viscosity were not the sole parameters affecting the creaming behavior of the TPS emulsions. They should be combined together to evaluate the stability of TPS emulsions. For the commercial TPS, all of them exhibited their optimum emulsion stabilizing capacity at pH 6 with the lowest instability index. This indicated that both the acidic and alkaline environments affected the creaming stability, which might be attributed to a number of factors. First, with fewer negative charges at pH < 6, electrostatic attraction forces might be stronger than repulsive forces between the polysaccharide molecules. In this regard, the stabilizing capacity of polysaccharides would be lower under acidic conditions. Second, with lower charge densities, fewer binding sites on polysaccharides are available to interact with the interface layer and more intermolecular hydrogen bonds might be formed. If this attractive force is sufficiently strong, it will promote droplet flocculation [62]. Under alkaline environments, even though with high charge densities, emulsion drops were less stable as the viscosities of the continuous polysaccharide phases were lower, resulting in higher instability indexes. It could be concluded that the high viscosity property of TPS was a primary variable affecting the stability of the emulsion, while the electrostatic repulsive force among TPS emulsions also played an important but secondary role in stabilizing the system. Furthermore, from TMS to TPS-160, the emulsion stabilities at pH 6 were significantly different (*p* < 0.05) and showed an increasing trend. This was attributed to the molecular mass, which could affect the polysaccharides’ ability to stabilize oil drops by changing the solutions’ viscosity. What should be mentioned here is that the effect of the molecular mass on the stability of emulsions should be discussed specifically in the case of polysaccharides from the same origin. Thus, it was meaningless to compare L-TPS emulsion’ stability with those prepared by commercial TPS based on their molecular masses. Another phenomenon observed was that the TMS emulsion showed higher stability compared to the L-TPS emulsion at pH 6, which was different from the results of their ESI values. This might be caused by the different methods used. The results of ESI values in our research mainly demonstrated the stability of emulsions at the initial stage (in 10 min), while the instability indexes were supposed to be more reliable to assess the long-term stability of emulsions.

Different from the trend of commercial TPS emulsions, the instability index of L-TPS emulsions increasing from pH 2 to 10 revealed that acidic environments could effectively avoid the flocculation of coated oil droplets. This result may be attributed to the proteins in the L-TPS samples. At low pH conditions, proteins with positive charges could interact with polysaccharides with negative charges and form protein-polysaccharide complexes on the surface of oil drops [63]. Similar phenomena were observed in the research of Tavernie et al. [64], where stable dispersions could be formed at pH 3 due to the good electrostatic interaction between SPI and κ-CG.

## 4. Conclusions

This study showed that the chemical compositions and macromolecular characteristics of the TPS extracted in our lab and commercial TPS were different. These TPS presented various emulsifying properties depending on their molecular characters, including molecular mass, monosaccharide composition, and protein content. With a high content of protein and a low molecular mass, L-TPS showed the best emulsifying activity. On the contrary, TPS-160 and TPS-120 had better capacities to stabilize emulsions compared to L-TPS. The pH had a significant effect on the properties of these TPS emulsions. All the commercial TPS emulsions had good stabilities at pH 6, where viscosity played a more important role than electrostatic repulsion in stabilizing the emulsion system. However, the stability of the L-TPS emulsion decreased with the pH increasing from 2 to 10 and possibly the electrostatic attraction between L-TPS and the proteins inside made the emulsion the most stable at pH 2. The result indicates that L-TPS has the potential to be used as a novel emulsifier, while commercial TPS, especially TPS-160, can be used as emulsion-stabilizing ingredients in the food industry. In summary, this work has discovered a new application area for TPS. As an ongoing investigation, the form of proteins in L-TPS and how these proteins affect the behavior of TPS on the surface of oil drops will be figured out.

## Figures and Tables

**Table 1:** Basic information of *Tremella fuciformis* polysaccharides.

Samples	Peak Molecular Mass (× 10^6^ g/mol)	Total Carbohydrate (%)	Moisture Content (%)	Ash Content (%)	Protein Content (%)
L-TPS	17.54 ± 0.23 ^d^	72.67 ± 2.55 ^b^	12.22 ± 0.12 ^b^	6.25 ± 0.05 ^a^	2.33 ± 0.19 ^b^
TMS	13.38 ± 1.39 ^c^	80.99 ± 2.07 ^a^	7.69 ± 0.14 ^a^	5.79 ± 0.08 ^a^	0.73 ± 0.02 ^a^
TPS-120	29.94 ± 3.30 ^b^	78.86 ± 1.06 ^a^	8.16 ± 0.03 ^a^	6.86 ± 0.07 ^a^	0.87 ± 0.01^a^
TPS-160	41.68 ± 5.12 ^a^	79.02 ± 0.95 ^a^	7.80 ± 0.03 ^a^	5.84 ± 0.02 ^a^	0.80 ± 0.002 ^a^

Results are means ± standard deviations of three replicates. Superscripts with different letters in same column indicate significant differences (*p* < 0.05).

**Table 2:** Monosaccharide composition of *Tremella fuciformis* polysaccharides (%).

Sample	Fuc	Gal	Glc	Xyl	Man	Gal-A	Glc-A
L-TPS	13.17 ± 1.17 ^b^	0.96 ± 0.12 ^b^	14.98 ± 3.33 ^b^	14.92 ± 3.89 ^a^	32.07 ± 3.71 ^b^	0.13 ± 0.01 ^b^	25.38 ± 2.98 ^b^
TMS	19.26 ± 2.71 ^a^	0.55 ± 0.13 ^a^	4.29 ± 0.64 ^a^	16.51 ± 3.19 ^a^	44.83 ± 5.94 ^a^	0.13 ± 0.01 ^b^	14.44 ± 1.22 ^a^
TPS120	20.00 ± 1.44 ^a^	0.44 ± 0.20 ^a^	3.31 ± 0.88 ^a^	18.08 ± 2.99 ^a^	44.91 ±3.12 ^a^	0.41 ± 0.19 ^a^	12.85 ± 1.34 ^a^
TPS160	19.15 ± 1.65 ^a^	1.00 ± 0.14 ^b^	5.49 ± 1.73 ^a^	16.74 ± 1.02 ^a^	43.70 ± 4.29 ^a^	0.22 ± 0.02 ^a^	13.70 ± 2.04 ^a^

Results are means ± standard deviations of three replicates. Superscripts with different letters in same column indicate significant differences (*p* < 0.05). Abbreviations: Fuc: Fucose; Gal: galactose; Glc: glucose; Xyl: xylose; Man: mannose; Gal-A: galacturonic acid; and Glc-A: glucuronic acid.

**Table 3:** Droplet size of emulsions prepared by *Tremella fuciformis* polysaccharides (µm).

Sample		pH 2	pH 4	pH 6	pH 8	pH 10
L-TPS	d_3,2_	8.92 ± 0.03 ^a^	9.90 ± 0.16 ^b^	4.65 ± 0.03 ^c^	4.71 ± 0.03 ^c^	3.84 ± 0.02 ^d^
	d_4,3_	23.40 ± 0.40 ^a^	20.70 ± 0.06 ^b^	20.30 ± 0.06 ^d^	17.70 ± 2.00 ^c^	10.90 ± 0.06 ^d^
TMS	d_3,2_	22.20 ± 0.53 ^a^	15.10 ± 0.00 ^b^	5.45 ± 0.01 ^c^	4.41 ± 0.04 ^d^	4.27 ± 0.02 ^d^
	d_4,3_	125.00 ± 7.0 ^a^	37.20 ± 0.00 ^b^	11.70 ±0.06 ^c^	11.70 ± 0.06 ^c^	11.60 ± 0.06 ^c^
TPS-120	d_3,2_	58.70 ± 0.06 ^a^	13.20 ± 0.06 ^b^	4.41 ± 0.06 ^c^	3.50 ± 0.01 ^d^	3.63 ± 0.00 ^e^
	d_4,3_	78.60 ± 0.06 ^a^	32.50 ± 0.06 ^b^	11.30 ± 0.00 ^c^	10.30 ± 0.00 ^d^	10.10 ± 0.00 ^e^
TPS-160	d_3,2_	30.10 ± 0.44 ^a^	13.80 ± 0.26 ^b^	5.57 ± 0.06 ^c^	4.29 ± 0.05 ^d^	4.62 ± 0.01 ^d^
	d_4,3_	90.40 ± 2.26 ^a^	32.80 ± 2.83 ^b^	12.00 ± 0.06 ^c^	14.90 ± 2.01 ^c^	11.80 ± 0.00 ^c^

Results are means ± standard deviations of three replicates. Superscripts with different letters in same row indicate significant differences (*p* < 0.05).

**Table 4:** Flow consistency index (K) and flow behavior index (n) determined with power law equation, Equation (1), for homogeneous TPS solutions and TPS emulsions with the shear rate ranging from 0.1 to 100 s^−1^ at T = 25 °C.

Sample		pH 2	pH 4	pH 6	pH 8	pH 10
L-TPS	K (Pa·s^n^)	0.031 ± 0.009 ^a^	0.244 ± 0.002 ^b^	0.318 ± 0.012 ^c^	0.305 ± 0.004 ^c^	0.256 ± 0.051 ^b^
	n	0.851 ± 0.046 ^b^	0.695 ± 0.013 ^a^	0.642 ± 0.021 ^a^	0.667 ± 0.006 ^a^	0.680 ± 0.016 ^a^
	R^2^	0.999	0.998	0.998	0.997	0.998
TMS	K (Pa·s^n^)	0.077 ± 0.012 ^a^	0.356 ± 0.032 ^b^	0.439 ± 0.010 ^d^	0.392 ± 0.014 ^c^	0.379 ± 0.018 ^bc^
	n	0.799 ± 0.018 ^b^	0.658 ± 0.024 ^a^	0.625 ± 0.031 ^a^	0.638 ± 0.065 ^a^	0.634 ± 0.030 ^a^
	R^2^	0.998	0.997	0.997	0.997	0.996
TPS-120	K (Pa·s^n^)	0.271 ± 0.039 ^a^	0.513 ± 0.070 ^b^	0.772 ± 0.029 ^d^	0.652 ± 0.045 ^c^	0.599 ± 0.045 ^bc^
	n	0.680 ± 0.026 ^b^	0.547 ± 0.023 ^a^	0.553 ± 0.041 ^a^	0.523 ± 0.011 ^a^	0.532 ± 0.013 ^a^
	R^2^	0.998	0.995	0.994	0.995	0.994
TPS-160	K (Pa·s^n^)	0.365 ± 0.039 ^a^	0.830 ± 0.039 ^b^	0.973 ± 0.039 ^c^	0.850 ± 0.039 ^b^	0.856 ± 0.039 ^b^
	n	0.606 ± 0.016 ^b^	0.461 ± 0.011 ^a^	0.451 ± 0.009 ^a^	0.470 ± 0.020 ^a^	0.453 ± 0.021 ^a^
	R^2^	0.996	0.991	0.991	0.991	0.990
L-TPS emulsion	K (Pa·s^n^)	0.005 ± 0.002 ^a^	0.055 ± 0.006 ^b^	0.039 ± 0.002 ^c^	0.038 ± 0.005 ^c^	0.040 ± 0.003 ^c^
	n	0.920 ± 0.020 ^b^	0.838 ± 0.007 ^a^	0.844 ± 0.029 ^a^	0.844 ± 0.011 ^a^	0.839 ± 0.012 ^a^
	R^2^	0.994	0.987	0.999	0.999	0.999
TMS emulsion	K (Pa·s^n^)	0.172 ± 0.010 ^a^	0.023 ± 0.010 ^b^	0.053 ± 0.004 ^d^	0.041 ± 0.001 ^c^	0.037 ± 0.003 ^c^
	n	0.617 ± 0.021 ^a^	0.878 ± 0.011 ^d^	0.835 ± 0.008 ^c^	0.836 ± 0.015 ^c^	0.778 ± 0.017 ^b^
	R^2^	0.730	0.989	0.999	0.999	0.999
TPS-120 emulsion	K (Pa·s^n^)	0.589 ± 0.033 ^d^	0.067 ± 0.012 ^a^	0.182 ± 0.007 ^c^	0.165 ± 0.003 ^b^	0.188 ± 0.003 ^c^
	n	0.423 ± 0.025 ^a^	0.815 ± 0.014 ^c^	0.686 ± 0.015 ^b^	0.693 ± 0.005 ^b^	0.680 ± 0.010 ^b^
	R^2^	0.998	0.980	0.998	0.998	0.998
TPS-160 emulsion	K (Pa·s^n^)	1.136 ± 0.075 ^c^	0.338 ± 0.003 ^b^	0.270 ± 0.017 ^a^	0.242 ± 0.027 ^a^	0.262 ± 0.003 ^a^
	n	0.359 ± 0.025 ^a^	0.582 ± 0.016 ^b^	0.630 ± 0.001 ^c^	0.665 ± 0.005 ^d^	0.636 ± 0.013 ^c^
	R^2^	0.997	0.994	0.996	0.997	0.997

Results are means ± standard deviations of three replicates. Superscripts with different letters in same row indicate significant differences (*p* < 0.05).

## Data Availability

The data presented in this study are available in the article.

## References

[1] Coviello T., Matricardi P., Marianecci C., Alhaique F. (2007). Polysaccharide hydrogels for modified release formulations. J. Control. Release.

[2] Dickinson E. (2003). Hydrocolloids at interfaces and the influence on the properties of dispersed systems. Food Hydrocoll..

[3] Lal S.N., O’Connor C.J., Eyres L. (2006). Application of emulsifiers/stabilizers in dairy products of high rheology. Adv. Colloid Interface Sci..

[4] Ngouémazong E.D., Christiaens S., Shpigelman A., Van Loey A., Hendrickx M. (2015). The Emulsifying and Emulsion-Stabilizing Properties of Pectin: A Review. Compr. Rev. Food Sci. Food Saf..

[5] Chen X., Wu X., Zhang K., Sun F., Zhou W., Wu Z., Li X. (2022). Purification, characterization, and emulsification stability of high- and low-molecular-weight fractions of polysaccharide conjugates extracted from green tea. Food Hydrocoll..

[6] Li Q., Zhao T., Shi J., Xia X., Li J., Liu L., McClements D.J., Cao Y., Fu Y., Han L. (2022). Physicochemical characterization, emulsifying and antioxidant properties of the polysaccharide conjugates from Chin brick tea (Camellia sinensis). Food Chem..

[7] Kumari N., Kumar M., Radha, Lorenzo J.M., Sharma D., Puri S., Pundir A., Dhumal S., Bhuyan D.J., Jayanthy G. (2022). Onion and garlic polysaccharides: A review on extraction, characterization, bioactivity, and modifications. Int. J. Biol. Macromol..

[8] Olawuyi I.F., Park J.J., Hahn D., Lee W.Y. (2022). Physicochemical and Functional Properties of Okra Leaf Polysaccharides Extracted at Different pHs. Chemistry.

[9] Wang S., Qu D., Zhao G., Yang L., Zhu L., Song H., Liu H. (2022). Characterization of the structure and properties of the isolating interfacial layer of oil–water emulsions stabilized by soy hull polysaccharide: Effect of pH changes. Food Chem..

[10] Gu Y.S., Decker E.A., McClements D. (2005). Influence of pH and carrageenan type on properties of β-lactoglobulin stabilized oil-in-water emulsions. Food Hydrocoll..

[11] Xu X., Luo L., Liu C., McClements D.J. (2017). Utilization of anionic polysaccharides to improve the stability of rice glutelin emulsions: Impact of polysaccharide type, pH, salt, and temperature. Food Hydrocoll..

[12] Hu J., Lin S., Tan B.K., Hamzah S.S., Lin Y., Kong Z., Zhang Y., Zheng B., Zeng S. (2018). Photodynamic inactivation of Burkholderia cepacia by curcumin in combination with EDTA. Food Res. Int..

[13] Zhu Z., Hu J., Chen J., Xu W., Wang P., Kong L., Zheng B., Lin S. (2016). The promotion effect of polysaccharide isolated from fresh tremella on the growth of bifidobacteria. Adv. J. Food Sci. Technol..

[14] Cho E.J., Hwang H.J., Kim S.W., Oh J.Y., Baek Y.M., Choi J.W., Bae S.H., Yun J.W. (2007). Hypoglycemic effects of exopolysaccharides produced by mycelial cultures of two different mushrooms Tremella fuciformis and Phellinus baumii in ob/ob mice. Appl. Microbiol. Biotechnol..

[15] Wu Y.-J., Wei Z.-X., Zhang F.-M., Linhardt R.J., Sun P.-L., Zhang A.-Q. (2019). Structure, bioactivities and applications of the polysaccharides from Tremella fuciformis mushroom: A review. Int. J. Biol. Macromol..

[16] Zhang J., Zhang Y.-K., Liu Y., Wang J.-H. (2019). Emulsifying Properties of Tremella Fuciformis: A Novel Promising Food Emulsifier. Int. J. Food Eng..

[17] Hou F., Yang S., Ma X., Gong Z., Wang Y., Wang W. (2022). Characterization of Physicochemical Properties of Oil-in-Water Emulsions Stabilized by *Tremella fuciformis* Polysaccharides. Foods.

[18] Akyüz A., Ersus S. (2021). Optimization of enzyme assisted extraction of protein from the sugar beet (Beta vulgaris L.) leaves for alternative plant protein concentrate production. Food Chem..

[19] Staub A. (1965). Removeal of protein-Sevag method. Methods Carbohydr. Chem..

[20] DuBois M., Gilles K.A., Hamilton J.K., Rebers P.A., Smith F. (1956). Colorimetric method for determination of sugars and related substances. Anal. Chem..

[21] IDF (2001). Milk-Determination of nitrogen content-Part 1: Kjeldahl method. IDF.

[22] Vilar E.G., Ouyang H., O’Sullivan M.G., Kerry J.P., Hamill R., O’Grady M.N., Mohammed H.O., Kilcawley K.N. (2020). Effect of salt reduction and inclusion of 1% edible seaweeds on the chemical, sensory and volatile component profile of reformulated frankfurters. Meat Sci..

[23] McCarthy W.P., Blais H.N., O’Callaghan T.F., Hossain M., Moloney M., Danaher M., O’Connor C., Tobin J.T. (2022). Application of nanofiltration for the removal of chlorate from skim milk. Int. Dairy J..

[24] Belghith-Fendri L., Chaari F., Ben Jeddou K., Kallel F., Bouaziz F., Helbert C.B., Abdelkefi-Mesrati L., Ellouz-Chaabouni S., Ghribi-Aydi D. (2018). Identification of polysaccharides extracted from pea pod by-products and evaluation of their biological and functional properties. Int. J. Biol. Macromol..

[25] Klein M., Aserin A., Svitov I., Garti N. (2010). Enhanced stabilization of cloudy emulsions with gum Arabic and whey protein isolate. Colloids Surf. B.

[26] Li P., Jiang Z., Sun T., Wang C., Chen Y., Yang Z., Du B., Liu C. (2018). Comparison of structural, antioxidant and immuno-stimulating activities of polysaccharides from Tremella fuciformis in two different regions of China. Int. J. Food Sci..

[27] Wang L., Zhang B., Xiao J., Huang Q., Li C., Fu X. (2018). Physicochemical, functional, and biological properties of water-soluble polysaccharides from Rosa roxburghii Tratt fruit. Food Chem..

[28] Liu J., Meng C.-G., Yan Y.-H., Shan Y.-N., Kan J., Jin C.-H. (2016). Structure, physical property and antioxidant activity of catechin grafted Tremella fuciformis polysaccharide. Int. J. Biol. Macromol..

[29] Fadavi G., Mohammadifar M.A., Zargarran A., Mortazavian A.M., Komeili R. (2014). Composition and physicochemical properties of Zedo gum exudates from Amygdalus scoparia. Carbohydr. Polym..

[30] Sheng Z., Liu J., Yang B. (2021). Structure Differences of Water Soluble Polysaccharides in *Astragalus membranaceus* Induced by Origin and Their Bioactivity. Foods.

[31] Wen L., Gao Q., Ma C.-W., Ge Y., You L., Liu R.H., Fu X., Liu D. (2016). Effect of polysaccharides from Tremella fuciformis on UV-induced photoaging. J. Funct. Foods.

[32] Palacios I., García-Lafuente A., Guillamón E., Villares A. (2012). Novel isolation of water-soluble polysaccharides from the fruiting bodies of Pleurotus ostreatus mushrooms. Carbohydr. Res..

[33] Giavasis I. (2014). Bioactive fungal polysaccharides as potential functional ingredients in food and nutraceuticals. Curr. Opin. Biotechnol..

[34] Thakur M., Singh H.K. (2013). Mushrooms, their bioactive compounds and medicinal uses: A review. Med. Plants Int. J. Phytomed. Relat. Ind..

[35] Qun G., Ajun W. (2006). Effects of molecular weight, degree of acetylation and ionic strength on surface tension of chitosan in dilute solution. Carbohydr. Polym..

[36] Wang D., Wang D., Yan T., Jiang W., Han X., Yan J., Guo Y. (2019). Nanostructures assembly and the property of polysaccharide extracted from Tremella Fuciformis fruiting body. Int. J. Biol. Macromol..

[37] Huang T.-Y., Yang F.-L., Chiu H.-W., Chao H.-C., Yang Y.-J., Sheu J.-H., Hua K.-F., Wu S.-H. (2022). An Immunological Polysaccharide from *Tremella fuciformis*: Essential Role of Acetylation in Immunomodulation. Int. J. Mol. Sci..

[38] Singh B., Pal L. (2012). Sterculia crosslinked PVA and PVA-poly (AAm) hydrogel wound dressings for slow drug delivery: Mechanical, mucoadhesive, biocompatible and permeability properties. J. Mech. Behav. Biomed. Mater..

[39] Vidal C.A.G. (2013). Molecular Weight Effects in Guar Gum Adsorption and Depression of Talc. Ph.D. Thesis.

[40] Singh B., Sharma V., Chauhan D. (2010). Gastroretentive floating sterculia–alginate beads for use in antiulcer drug delivery. Chem. Eng. Res. Des..

[41] Yuen S.-N., Choi S.-M., Phillips D.L., Ma C.-Y. (2009). Raman and FTIR spectroscopic study of carboxymethylated non-starch polysaccharides. Food Chem..

[42] Chiovitti A., Bacic A., Craik D.J., Munro S.L., Kraft G.T., Liao M.-L. (1997). Cell-wall polysaccharides from Australian red algae of the family Solieriaceae (Gigartinales, Rhodophyta): Novel, highly pyruvated carrageenans from the genus Callophycus. Carbohydr. Res..

[43] De Baets S., Vandamme E.J. (2001). Extracellular Tremella polysaccharides: Structure, properties and applications. Biotechnol. Lett..

[44] Alba K., Kontogiorgos V. (2017). Pectin at the oil-water interface: Relationship of molecular composition and structure to functionality. Food Hydrocoll..

[45] Mirmoghtadaie L., Aliabadi S.S., Hosseini S.M. (2016). Recent approaches in physical modification of protein functionality. Food Chem..

[46] Kontogiorgos V. (2019). Polysaccharides at fluid interfaces of food systems. Adv. Colloid Interface Sci..

[47] Xu Q.-X., Shi J.-J., Zhang J.-G., Li L., Jiang L., Wei Z.-J. (2016). Thermal, emulsifying and rheological properties of polysaccharides sequentially extracted from Vaccinium bracteatum Thunb leaves. Int. J. Biol. Macromol..

[48] Shi J.-J., Zhang J.-G., Sun Y.-H., Qu J., Li L., Prasad C., Wei Z.-J. (2016). Physicochemical properties and antioxidant activities of polysaccharides sequentially extracted from peony seed dreg. Int. J. Biol. Macromol..

[49] Costa A.L.R., Gomes A., Furtado G.D.F., Tibolla H., Menegalli F.C., Cunha R.L. (2020). Modulating in vitro digestibility of Pickering emulsions stabilized by food-grade polysaccharides particles. Carbohydr. Polym..

[50] Gómez-Mascaraque L.G., Martínez-Sanz M., Hogan S.A., López-Rubio A., Brodkorb A. (2019). Nano- and microstructural evolution of alginate beads in simulated gastrointestinal fluids. Impact of M/G ratio, molecular weight and pH. Carbohydr. Polym..

[51] Mirhosseini H., Tan C.P., Hamid N.S.A., Yusof S. (2008). Effect of arabic gum, xanthan gum and orange oil contents on ζ-potential, conductivity, stability, size index and pH of orange beverage emulsion. Colloids Surf. A Physicochem. Eng. Asp..

[52] Han Y., Cheng Z., Zhang Y., Zhang N., Zhu X., Chen X., Shao Y., Cheng Y., Wang C., Luo Y. (2020). Effect of metal ions and pH on the emulsifying properties of polysaccharide conjugates prepared from low-grade green tea. Food Hydrocoll..

[53] Qu D., Wang S., Zhao H., Liu H., Zhu D., Jiang L. (2021). Structure and interfacial adsorption behavior of soy hull polysaccharide at the oil/water interface as influenced by pH. Food Hydrocoll..

[54] Behrouzain F., Razavi S.M., Joyner H. (2020). Mechanisms of whey protein isolate interaction with basil seed gum: Influence of pH and protein-polysaccharide ratio. Carbohydr. Polym..

[55] Osano J.P., Hosseini-Parvar S.H., Matia-Merino L., Golding M. (2014). Emulsifying properties of a novel polysaccharide extracted from basil seed (Ocimum bacilicum L.): Effect of polysaccharide and protein content. Food Hydrocoll..

[56] Santiago J.S.J., Salvia-Trujillo L., Palomo A., Niroula A., Xu F., Van Loey A.M., Hendrickx M.E. (2018). Process-induced water-soluble biopolymers from broccoli and tomato purées: Their molecular structure in relation to their emulsion stabilizing capacity. Food Hydrocoll..

[57] Krägel J., Derkatch S., Miller R. (2008). Interfacial shear rheology of protein–surfactant layers. Adv. Colloid Interface Sci..

[58] Bourbon A., Pinheiro A., Ribeiro C., Miranda C., Maia J., Teixeira J., Vicente A. (2010). Characterization of galactomannans extracted from seeds of Gleditsia triacanthos and Sophora japonica through shear and extensional rheology: Comparison with guar gum and locust bean gum. Food Hydrocoll..

[59] Carneiro-Da-Cunha M.G., Cerqueira M.A., Souza B.W., Teixeira J.A., Vicente A.A. (2011). Influence of concentration, ionic strength and pH on zeta potential and mean hydrodynamic diameter of edible polysaccharide solutions envisaged for multinanolayered films production. Carbohydr. Polym..

[60] Sriprablom J., Luangpituksa P., Wongkongkatep J., Pongtharangkul T., Suphantharika M. (2019). Influence of pH and ionic strength on the physical and rheological properties and stability of whey protein stabilized o/w emulsions containing xanthan gum. J. Food Eng..

[61] Nakauma M., Funami T., Noda S., Ishihara S., Al-Assaf S., Nishinari K., Phillips G.O. (2008). Comparison of sugar beet pectin, soybean soluble polysaccharide, and gum arabic as food emulsifiers. 1. Effect of concentration, pH, and salts on the emulsifying properties. Food Hydrocoll..

[62] Paraskevopoulou A., Boskou D., Kiosseoglou V. (2005). Stabilization of olive oil-lemon juice emulsion with polysaccharides. Food Chem..

[63] Albano K.M., Cavallieri L.F., Nicoletti V.R. (2019). Electrostatic interaction between proteins and polysaccharides: Physicochemical aspects and applications in emulsion stabilization. Food Rev. Int..

[64] Tavernier I., Patel A.R., Van der Meeren P., Dewettinck K. (2017). Emulsion-templated liquid oil structuring with soy protein and soy protein: κ-carrageenan complexes. Food Hydrocoll..

